# The “*Journal of Functional Morphology and Kinesiology*” Journal Club Series: Highlights of Recent Papers in Pediatric Exercise

**DOI:** 10.3390/jfmk4020021

**Published:** 2019-04-12

**Authors:** Vito Pavone, Andrea Vescio, Gianluca Testa, Helmi Chaabene, Antonino Bianco

**Affiliations:** 1 Department of General Surgery and Medical Surgical Specialties, Section of Orthopedics and Traumatology, A.O.U. Policlinico-Vittorio Emanuele, University of Catania, Via Santa Sofia 78, 95123 Catania, Italy; 2 Division of Training and Movement Sciences, Research Focus Cognition Sciences, University of Potsdam, Am Neuen Palais 10, 14469 Potsdam, Germany; 3 Sport and Exercise Sciences Research Unit, University of Palermo, Via Giovanni Pascoli, 6, 90144 Palermo, Italy

## Abstract

We are glad to introduce the ninth Journal Club. This edition is focused on several relevant studies published in the last years in the field of Pediatric Exercise, chosen by our Editorial Board members and their colleagues. We hope to stimulate your curiosity in this field and to share with you the passion for the sport as seen also from the scientific point of view. The Editorial Board members wish you an inspiring lecture.

## 1. Introduction

Since the 1930s, when the first scientific study on physiological responses of children to exercise was published in Harvard [1], pediatric exercises have inspired the interest of researchers. Nevertheless, it was only in 1983, after the publication of the Oded Bar-Or book [2], Sports Medicine for the Practitioner, that the physiological responses aptitude to exercise in children was highlighted, stimulating the need to improve exercise habits at a young age and increase the quality of life in adulthood [3]. In the first years of life, unstructured physical play time allows the development of motor skills, promoting social interaction and creativity [4]. In childhood, several studies have suggested the physiological benefits of exercise regarding the maintenance of weight, bone metabolism stimulation, and the improvement of sports performance. Moreover, several scientific evidences have revealed numerous benefits in improving mental development, even if the mechanism is relatively unknown [4]. The UK guidelines on physical activity encourage 60-min or more of daily practice for children and the young population. Pediatric exercises that strengthen muscle and bone and promote flexibility should be performed at least three days per week [5]. Different studies have investigated the future prospects of pediatric exercise, for example, on the correlation between exercises and the benefits of molecular mechanisms or the possibility of developing exercises tailored for patients.

## 2. Recent Papers Regarding Pediatric Exercise

### 2.1. Role of Quadriceps Exercises in Child and Adolescent Population Affected by Patellar Instability

#### Highlight by Vito Pavone, Andrea Vescio, Gianluca Testa

Patellar dislocation is a common knee injury in the pediatric population, generally caused by a deficiency or a complete lesion of the medial patellofemoral ligament (MPFL). MPFL insufficiency causes a limitation of the patellofemoral joint, promoting cartilage tear and degeneration, with consequent early osteoarthrosis in young adulthood [6].

Although surgical reconstruction of MFPL, as the primary treatment of patellar dislocation, is widespread among orthopedic surgeons, conservative treatment, consisting of physical exercises and immobilization techniques, is generally indicated after the first episode of patellar dislocation. After a period of 2–3 weeks of orthosis or brace immobilization with 15 to 30 degrees of flexion [7], non-operative management consists of the optimization of the neuromusculoskeletal system, muscle recruitment and strengthening, (glutei, quadriceps, hamstring, and calf complex), providing dynamic stability [8]. In the literature, the reconditioning of the vastus medialis obliquus muscle is considered as the principal aim of physical therapy; in fact, it promotes successful healing of the MPFL, improving neuromusculoskeletal control, and preventing further dislocations [8]. Moreover, several physicians have described general quadriceps (GQ) exercises for rehabilitation after patellar dislocation or instability affected patients.

The study of Moiz et al. [8] described clinical outcomes after the nonoperative management of primary or recurrent patellar dislocations, focusing partially on physical exercises. A systematic review of the principal electronic databases (including MEDLINE, Embase, and the Cochrane Library) was performed by three independent authors. A total of 25 included studies was divided into four broad categories of nonoperative interventions based on immobilization, weight-bearing status, quadriceps exercise type, and alternative therapies. The authors compared the outcomes between GQ and vastus medialis specific (VMS) exercises and the combination of two exercises types. All of the evaluated studies highlighted the importance of early active rehabilitation; in fact, approximately 60% of patients demonstrated improvements in functional outcomes. According to the Lysholm scores, patients treated with GQ exercises reported better results than VMS with statistical significance. 

Physical exercises have optimal outcomes in children and adolescents after primary dislocation and are helpful in the prevention of recurrent patellar dislocations, but further a standardized physical protocol should be developed.

### 2.2. Adaptations to Resistance Training in Youth: Does the Maturity Level Matter?

#### Highlight by Helmi Chaabene

Resistance training has long been thought to be deleterious to the normal growth pattern in youth (e.g., epiphyseal plate damage) [9]. However, over the past two decades, strong evidence-based reports have shown plenty of advantages for resistance training from the health-, psychological-, and performance-related perspectives in youth when properly designed and administered [10,11,12,13]. It has generally been demonstrated that adaptations following resistance training in youth (e.g., pre-pubertal) are predominantly attributed to neural factors [14,15,16]. This is due to the lack of circulating androgens (e.g., testosterone) in the pre-pubertal age, among others, which are necessary to trigger morphological adaptations [13,17]. On the other hand, resistance training-related adaptations in post-pubertal individuals could be driven by both neural (e.g., motor unit recruitment, coding rate, etc.) and structural factors (i.e., muscle hypertrophy) [18]. Given the different training-related adaptation mechanisms, one might ask the question of whether there is a difference in resistance training-related adaptation magnitude between pre-pubertal and post-pubertal individuals. 

The study of Moran et al. [19] addressed this question. The authors examined and compared the effects of resistance training-related adaptations on measures of strength (i.e., handgrip peak force and isometric mid-thigh pull peak force) and power (i.e., vertical jump) between pre- and post-pubertal individuals. The strength of this study is that both experimental groups were compared with matched control groups to effectively account for changes due to maturation [19]. After eight weeks of training, results showed that strength and power were trainable to different degrees in pre-pubertal and post-pubertal individuals. More specifically, the authors demonstrated greater training-related adaptations in post- compared to pre-pubertal individuals. Moran et al. [19] attributed the lower magnitude of improvements to the fewer pathways of adaptation in the pre-pubertal group (i.e., mainly in the level of neural drive) compared to the post-pubertal group (i.e., neural and morphological factors). However, the underlying mechanisms leading to different training-related adaptations between pre-pubertal and post-pubertal individuals have to be further explored in a similarly designed study research.

### 2.3. Pediatric Exercise: Play Stronger! To Live Better and Longer

#### Highlight by Antonino Bianco

Pediatric exercise science is a quite recent research area characterized by a particular focus on human movement oriented to the proper growth and development of children. The expert’s choice 2018, recently published in PES Journal [20], represents an exciting update of the most advanced research in the field of pediatric exercise science. Of interest, the Avery Faigenbaum commentary indicates, once more, how important muscular strength is in the pediatric age for the capacity to determine lower rates of adiposity, cardiovascular disease, and metabolic risk factors. Ohuchi et al. recently published an interesting paper that also recommended pediatric exercise in the case of pediatric patients. The authors concluded that “*A positive exercise capacity trajectory during childhood predicts better adult Fontan pathophysiology, including better prognosis*” [21]. Moreover, Alon Eliakim in 2018 highlighted the relationship between hormones and pediatric exercise [22]. The author clearly discussed the impact of exercise training on IGF-I levels during the tapering periods and the different moments of the seasons of young athletes. Additionally, the influence of GH was also discussed. The author reported as follows: “*growth hormone administration elicits significant changes in body composition and possible limited effect on anaerobic performance but does not increase either muscle strength or aerobic exercise capacity in healthy, young subjects*” [22]. Marianna Alesi et al. in 2016 analyzed the effects of a football exercise program on motor skills and executive functions where they discovered improvements in visuo-spatial working memory, attention, planning, and inhibition after an exercise program administrated for a period of six months [23].

In this commentary, the multidimensional nature of pediatric exercise was highlighted. When human movements become organized, structured, and periodized, professionals can obtain impressive results in terms of proper growth, development, cognitive performance, skills, and wellbeing. The crucial points are always the same: intensity, frequency, volume, and duration of the training stimuli. A proper balance is necessary and muscular strength is always the key biomotor ability that determines all of the adaptations above-mentioned.
